# Can *Toxoplasma gondii* Pave the Road for Dementia?

**DOI:** 10.1155/2020/8859857

**Published:** 2020-07-30

**Authors:** Enas A. El Saftawy, Noha M. Amin, Rania M. Sabry, Noha El-Anwar, Rania Y. Shash, Eman H. Elsebaie, Rita M. Wassef

**Affiliations:** ^1^Medical Parasitology Department, Faculty of Medicine, Cairo University, Cairo, Egypt; ^2^Armed Forces College of Medicine, Cairo, Egypt; ^3^Pathology Department, Faculty of Medicine, Cairo University, Cairo, Egypt; ^4^Pathology Department, Faculty of Medicine, Tanta University, Egypt; ^5^Medical Microbiology and Immunology Department, Faculty of Medicine, Cairo University, Cairo, Egypt; ^6^Public Health and Community Medicine, Faculty of Medicine, Cairo University, Cairo, Egypt; ^7^Medical Parasitology Department, Faculty of Medicine, Helwan University, Cairo, Egypt

## Abstract

Dementia is an ominous neurological disease. Scientists proposed a link between its occurrence and the presence of *Toxoplasma gondii* (*T. gondii)*. The long-term sequels of anti-*Toxoplasma* premunition, chiefly dominated by TNF-*α*, on the neurons and their receptors as the insulin-like growth factor-1 receptor (IGF-1R), which is tangled in cognition and synaptic plasticity, are still not clear. IGF-1R mediates its action via IGF-1, and its depletion is incorporated in the pathogenesis of dementia. The activated TNF-*α* signaling pathway induces NF-*κβ* that may induce or inhibit neurogenesis. This study speculates the potential impact of anti-*Toxoplasma* immune response on the expression of IGF-1R in chronic cerebral toxoplasmosis. The distributive pattern of *T. gondii* cysts was studied in association with TNF-*α* serum levels, the in situ expression of NF-*κβ*, and IGF-1R in mice using the low virulent ME-49 *T. gondii* strain. There was an elevation of the TNF-*α* serum level (*p* value ≤ 0.004) and significant upsurge in NF-*κβ* whereas IGF-1R was of low abundance (*p* value < 0.05) compared to the controls. TNF-*α* had a strong positive correlation with the intracerebral expression of NF-*κβ* (*r* value ≈ 0.943, *p* value ≈ 0.005) and a strong negative correlation to IGF-1R (*r* value -0.584 and -0.725 for area% and O.D., respectively). This activated TNF-*α*/NF-*κβ* keeps *T. gondii* under control at the expense of IGF-1R expression, depriving neurons of the effect of IGF-1, the receptor's ligand. We therefore deduce that *T. gondii* immunopathological reaction may be a road paver for developing dementia.

## 1. Introduction

Dementia is a progressive neurodegenerative disease that is characterized by irreversible neuronal losses [1]. Dementia had been suggested a long time ago to be caused by the excessive constitution of amyloid protein that leads to cell death, synaptic dysfunction, and brain atrophy [2]. However, pharmacological trials conducted to test this theory have been unsuccessful, and on the contrary, most cases worsen especially in old age [3]. Interestingly, during the recent decades, interventional epidemiology in neuroscience has been introduced where the possible interaction between the pathogenesis of dementia and various individual/environmental factors is studied, and therefore, different therapeutic protocols will be determined for various patients' subgroups [4]. In this context, identifying new targets for this disease is a matter of urgency [5]. For instance, pathogenic agents, including *Toxoplasma gondii* (*T. gondii*) [6], *Herpes simplex virus*-1 [7], and *Chlamydia pneumoniae* [8], were thought to be implicated in the pathogenesis of some neurodegenerative diseases.

According to the Centre for Diseases Control (CDC), toxoplasmosis is thought to be a foodborne and cosmopolitan infection that exceeds 60% in some populations [9]. Despite being of high prevalence worldwide, variations of its incidence from one geographic area to another are assumed to be caused by various environmental and lifestyle factors [10]; however, many studies have implicated the sexual replication of *T. gondii* and the prevalence of oocysts in feline feces as a chief cause [9, 11].

*T. gondii* is considered to be among the most resistant intracellular parasites but is thought to remain asymptomatic in most cases. However, recent human studies have implicated toxoplasmosis as a hidden contributor to various forms of dementia [12–17].

Although *T. gondii* exists in various organs in the human body, the parasite is chiefly prevalent in the central nervous system [12, 13]. In low-virulence strains, parasites transform into a cyst stage, and despite the debate concerning its preferable distributive pattern, it had been demonstrated in the amygdala and the hippocampus [18]. The latter areas are two medial temporal lobe structures that act in concert to allow flexible cognitive performance. The amygdala modulates the programming process of hippocampal-dependent memories, while the hippocampus stores the emotional symbols of events to affect the amygdala's responses. Indeed, this combination aids in the matching, generation, and flexible usage of acquired information [19, 20].

Studies of the immune microenvironment in the acute phase of lethal-strain toxoplasmosis have revealed that immunity against intracellular replicating tachyzoites relies crucially on a recognizable surge in the cytokine panel secreted by both Th1 and CD8+ cells, including TNF-*α*, Il-12, and IFN-*γ* [21]. According to experimental models, this type of immunity fails to kill the parasite but causes host cell lysis, massive neurodegenerative foci, and death [22]. and death [22]. In contrast, T. gondii avirulent (or non-virulent) strain induces a latent courseIn contrast, *T. gondii*, gondii avirulent (or non-virulent) strain induces a latent course IL-10 and TGF-*β* and modest levels of Th1 cytokine (chiefly TNF-*α*) and inhibited nitric oxide (NO). This immune regulatory mode, known as premunition immunity, allows the survival of both the host cells and the parasite [19, 23].

Tumour necrosis factor-alpha (TNF-*α*) is a cytokine that is well-recognized for its crucial role in the signaling pathway of the nervous system and has been identified in the pathogenesis of multiple inflammatory diseases [24]. Although TNF-*α* is mainly associated with neurotoxicity, it has also been found to have a neuroprotective effect [25, 26]. However, persistent seropositive levels of TNF-*α* have been recognized as a mediator of cachexia in chronic inflammatory conditions (henceforth its synonym cachectin or cachexin) [27].

Functions of TNF-*α* involve the induction of the nuclear factor-kappa beta (NF-*κβ*) signaling pathway, a process that requires the translocation of NF-*κβ* DNA binding factor (p65 (RelA)) from the cell cytoplasm to the nucleus [28–30]. In turn, NF-*κβ* is a vital proinflammatory transcription factor that controls the initiation of the innate and adaptive immune cascades [31], as it attributes in (1) the transcription of the inflammatory cytokines [32]; (2) the differentiation, proliferation, survival [33], and maturation of the immune cells [34–36]; (3) the differentiation of CD8+ T cells into both effector [36, 37] and memory cells [38]; (4) the mediation of the signaling pathway in Tregs [34, 39–41]; and (5) the induction of anti-/proapoptotic signals in regard to the nature of the stimuli [42–45].

NF-*κβ* is involved also in the cerebral aging processes [46], damage of white matter, and impairment of cognition (in the murine experimental model) [47]. However, its suppression induces apoptosis since it is involved in BCL-2 activation in lymphoma [48].

Interestingly, in a strain-dependent manner, activation of NF-*κβ* was found to occur via the apical secretory proteins of the parasite and thus the host cell death and inflammation [49], and on the contrary, in some virulent strains, the nuclear translocation of NF-*κβ* had been shown to be blocked via the degradation of p65 [50]. Amusingly, it had been deduced that the parasite tends to concentrate the NF-*κβ* inhibitory protein (phosphorylated I*κβ* protein) in its parasitophorous vacuole that would induce antiapoptotic genes in the parasite and promote its survival and replication [51].

Several authors deduced the interaction of *T. gondii* with various host receptors during its establishment [52–54], where some of them were incorporated into psychological disorders [55]. Still, little is known about the expression of insulin-like growth factor-1 receptor (IGF-1R) during toxoplasmosis. IGF-1R is a heterotetrameric glycoprotein structure composed of two *α* and two *β* subunits [56] that had been recognized for its neurotrophic influence in the neuronal regeneration [57]. IGF-1R signaling is involved in (1) the lipid and glucose metabolism by autocrine and paracrine mechanisms (anabolic functions) [58, 59], (2) the promotion of brain growth and development, (3) the unison of critical neuroprotective strategies [60] and some antiapoptotic functions [59], and (4) the reduction of the cerebral amyloid-*β* level [61]. IGF-1R was described as an analog to the insulin hormone receptor with more potent growth-stimulating activity [62].

IGF-1R mRNA expression is promoted by its ligand (IGF-I), which binds to the extracellular *α* subunits of IGF-1R and initiates the autophosphorylation of the 3-tyrosine residues in the kinase domain of the *β* subunits [63, 64].

In human studies, low serum levels of the IGF-1R ligand have been associated with increased risks of Alzheimer's and lower brain volume (cerebral atrophy), and higher serum levels of IGF-1R ligand are considered as a safeguard against various clinical and subclinical presentations of neurological degeneration [65–69].

The main goal of this study was to reveal the possible unrecognizable influence of anti-*Toxoplasma* premunition immunity in creating a climate favorable to induce dementia using an experimentally accessible murine model. In this regard, we reinvestigated the chronic cerebral infection in association with the alterations in the inflammatory cytokine (TNF-*α*), the transcription factor (NF-*κβ*), and the fine refinement between them and the expressive pattern of the neurotropic protein receptor (IGF-1R) in the cerebral memory centers (the amygdala and hippocampus).

## 2. Material and Methods

### 2.1. Animals and Experimental Design

Due to the difficulty of human cerebral sampling, the present study utilized murine cerebral tissues as a substitutive model. This study used unisex (male) Swiss albino immune-competent mice that were 9–11 weeks of age with an average weight of 30–35 gm. The experimental requirements were supplied by the Laboratory Animal House of the Parasitology Department, Theodor Bilharz Research Institute, Egypt. Housing conditions included a balanced diet formula, sanitary conditions, temperature control (22°C), regular light pattern (12 h light and 12 h dark), and suitable degree of humidity. Serological tests were performed periodically by the research unit to confirm that the murine models were clear of common pathogens such as murine viral hepatitis. All animal procedures were accepted by the Institutional Animal Care and Use Committee, Cairo University, and were recorded by CU/III/F/52/19 following the “Guidelines for the Care and Use of Laboratory Animals.” The mice were divided into two groups of six mice. Group 1 carried the parasitic infection while Group 2 was used as the healthy study control.

### 2.2. Parasite Inoculation

Cysts of *T. gondii* strain ME-49 were obtained from the National Research Center, Dokki, Giza, Egypt. The cysts used in the experiment were acquired from mice killed by cervical dislocation seven or more weeks after being infected. Each mouse brain was extracted manually in a sterile Falcon tube supplied with a Teflon pestle and a tissue grinder. Suspensions of brain homogenates were then prepared by adding 2 ml of Hanks' balanced salt solution. Using a light microscope with 40x magnification power, a 20 *μ*l sample was inspected in a wet mount for counting cysts. Each cyst in a 20 *μ*l sample corresponded to 100 cysts in the 2 ml suspension, which was the detective limit for the infective sample. In this study, the infection was initiated by administrating a suspension of 0.25 ml of 0.9% NaCl normal saline solution containing 15–20 cysts of the low-virulence ME-49 strain by the peroral route. The infected mice's brains containing tissue cysts were harvested on the twelfth week by cervical dislocation. Since the study used a cytogenic chronic strain, classical clinical manifestations of acute toxoplasmosis such as lethargy, coarse rough fur, and arched posture were not considered.

### 2.3. Quantification of the Parasite

This was performed by imaging each brain area per mouse using Olympus microscopy (10x eye piece × 4x objective lens) and hematoxylin and eosin tissue-cut sections. Images were inserted in the ImageJ software, where the grid tool was used such to allocate and enumerate the parasites within definite squares, and the pixel area per point (the grid's squares) was automatically calculated. The parasites were manually selected, and the counts were logged onto the image by the software directly. Areas where the parasites' and host cells' nuclei cannot be differentiated were dismissed. The mean number of cysts (*C*/*N*) and the mean pixel area (*A*/*N*) were obtained to be calculated as the average no. of cysts per *pixel*^2^ using the following equation [70–72]:

(1) $$\begin{array}{c} \frac{C/N}{A/N}=\frac{C}{A} \end{array}$$

where *C* is the no. of cysts, *N* is the number of manipulated squares, and *A* is the area per point.

### 2.4. Serology

*In vivo* production of TNF-*α* was quantified using the ELISA sandwich technique in the collected serum samples in the twelfth week after infection initiation. The MBS590025 ELISA kit (purchased from MyBioSource) was used due to its insignificant crossreactivity with TGF, GM-CSF, MCP-3, M-CSF, EGF MCP-1, IL-1, IL-16, and IL-8 and its sensitivity at minimal doses of 0–2.4 pg/ml. The procedure quantified the antigen under investigation between two coats of antibodies reactive to different epitopes. The steps of the procedure were as follows: the immobilization of the specific capture (polyclonal) antibody on a high protein-binding multiwell plate, the addition of test sera (Ag) to the wells, the application of the secondary antibody constituting the indicator system, and, finally, the incubation of the tested samples overnight at 37°C and in 5% CO_2_ for 30 min. The absorbance of the secondary antibody (optical density, O. D.) was measured by a spectrophotometer in parallel with a standard curve to determine the quantity of TNF-*α* (pg/ml) [73].

### 2.5. Immunohistochemistry

*Processing of cerebral tissues*: the tissues were first collected from both diseased and normal (control) hosts. Then, the tissue samples were preserved using 10% neutral buffered formalin (24 hours) to be hardened and to fixate the proteins and the vital structures within the cerebral tissues. Thereafter, tissues were embedded into paraffin wax to be processed into blocks ready for sectioning and mounting. Tissue-cut sections (5 *μ*m) of the processed paraffin blocks were cut to be stained with hematoxylin and eosin and IHC.

*Process of immune staining*: the expression of IGF-1R and NF-*κβ* was identified using primary monoclonal antibodies of murine origin: #CN: 414 A and C and #CN: E-AB-32232, respectively. For mouse-on-mouse (MOM) staining and kit staining, tissue-cut sections were incubated for 10 min in the hydrogen peroxide blocking solution to hinder endogenous peroxidase enzyme activity, followed by Ultra V Block. Thus, nonspecific background staining was avoided. A primary antibody at a dilution of 1 : 200 from the stock was then applied to tissues to identify the expression of the cellular membrane and the cytoplasmic IGF-1R protein by binding to its domains or by binding to the p65 subunit of NF-*κβ*. The antibody was diluted with 20 mmol/l TBS, pH 7.4 (10 mmol/l CaCl_2_, 0.1% NaN_3_, and 1% BSA) based on dilution experiments. The tissue sections were incubated overnight in the diluted antibody.

A biotinylated goat antipolyvalent secondary antibody was incubated afterward with the tissue-cut sections for 10 minutes at room temperature to bind with the primary antibody. The final staining was done in diaminobenzidine tetrahydrochloride (DAB) solution (49 ml TBS-buffer, 34 mg imidazole, 17 *μ*l 30% hydrogen peroxide, and 1 ml 30% DAB), for 5–15 min. The slides were washed with distilled water, with 70% ethanol for 1 min, and then again with distilled water. The nuclei were counterstained with Mayer's hematoxylin for 30 seconds. The extra stain was washed away with tap water. The slides were then transferred through an ascending ethanol series and xylene before mounting. EconoTek HRP Anti-Polyvalent (DAB) kits were obtained from ScyTek Laboratories, Logan, Utah, USA (#AEX080). Each step in the process of immunohistochemical staining was followed by three to four PBS washes at room temperature for eight minutes each. The full procedure was performed in regard to the manufacturer's guide.

### 2.6. Real-Time Quantitative Morphocytometry

Using a Leica Qwin 500 Image Analyzer (LEICA Imaging System Ltd., Cambridge, England), we conducted digital real-time quantitative photocytometry during the pathological and real-time quantitative morphometric analysis. The area percentage (area %: the percentage of positive expression in 10 fields) and the optical density (O.D.: the intensity of the stain) were measured and automatically calculated at 40x and 100x in 10 fields in each slide. The recorded values were then saved to be statistically analyzed [74].

### 2.7. Statistical Methods

The data were compiled in Microsoft Excel 2013 and analyzed using the Statistical Package for the Social Sciences (SPSS), version 24. Simple descriptive statistics (median and interquartile range) were used to summarize the skewed quantitative data, and frequencies were used for the qualitative data. We used the Mann–Whitney *U* test and Spearman's *ρ* correlation to compare the abnormally distributed quantitative variables. The ANOVA test was performed where *p* values less than 0.05 were considered statistically significant. *r*values (positive or negative according to the sign) less than 0.5 were considered to show weak correlation, values from 0.5 to 0.7 showed a moderate correlation, and *r*values above 0.7 showed strong correlation [75–77].

## 3. Results

### 3.1. The Distribution of *T. gondii* Cysts in the Brain of the Infected Mice

**Figure 1:**
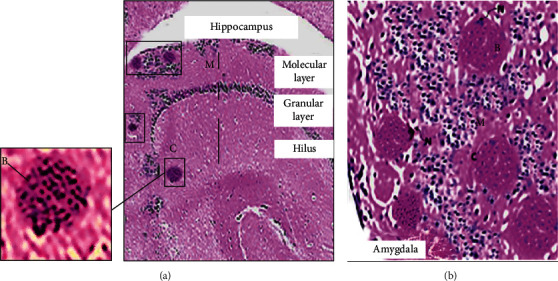
Hematoxylin and eosin stain where the plate reflects the cerebral lesions of *T. gondii* in the chronically infected Swiss albino mice. (a) Hippocampus with scattered variable-sized bradyzoites encased in cysts (C) in the hilus layer and the molecular layer (M) of the ventral hippocampus (40x). (b) Amygdala lodged with hundreds of bradyzoites (B) in cysts (note the basophilic dot-like terminal nuclei of the parasite) encased in the intracellular cysts (C) (note the lateral nucleus of the host cell (N)) surrounded by mononuclear chronic inflammatory infiltrates (M) (200x).

*T. gondii* was capable of being incorporated into the cerebral tissues in the form of a cystic stage as shown in Figure 1, which is remarkable for the chronicity of the parasite. The lesions were in the form of clusters of cysts with well-defined cyst walls and hundreds of bradyzoites. The parasitized cerebral cells were noted to be embedded among the surrounded neurons. The examined specimens were compared with the healthy control and did not show any histological changes except for some inflammatory infiltrates. Since isolation of the parasite was not performed, micrometry was not recommended to avoid false results in measurements.

**Figure 2:**
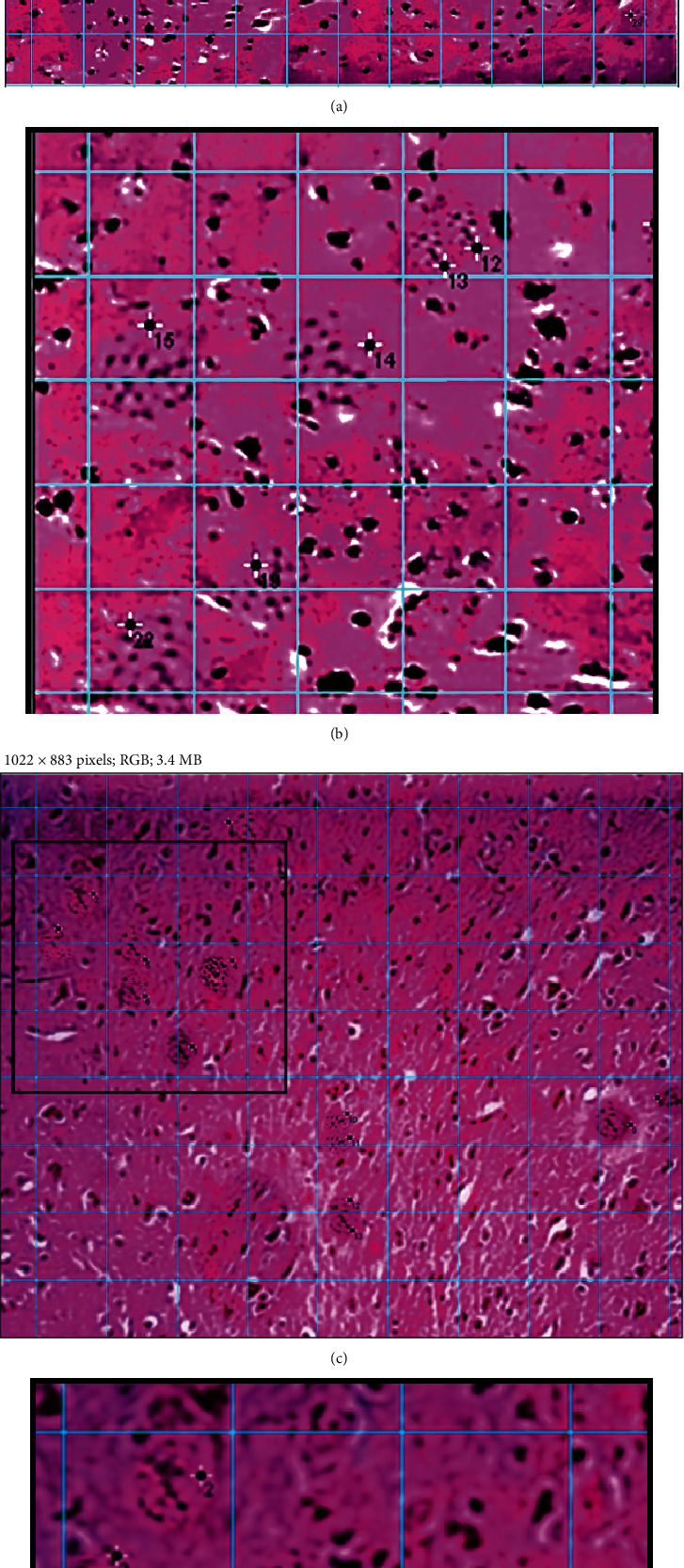
Estimation process of the *T. gondii* cysts in brain tissue-cut sections stained with H&E in a magnification power (40x). Quantification was based on creating an image grid (in blue colour) with the ImageJ software and manual selection of the parasites (asterisk with numbers) within the boundaries of the grid. (a) A partial hippocampus region with clusters of cysts (28 cysts), and (c) shows separate cysts (13 cysts) in the amygdala region. Note that (b, d) are cropped magnified portions from (a) and (c), respectively.

The ratios of the mean weights (counts) of the cysts to the mean pixel area of the hippocampus and amygdala tissue-cut sections were of insignificant differences (*p* value > 0.05) (Figure 2) while yielding *p* values less than 0.05, i.e., significantly different values when compared with other brain areas. Based on the acceptance to consider structural alterations in the hippocampus and amygdala grey matter as diagnostic tools for dementia in previous experimental models [78, 79], in addition to the primary results of the parasite's ratios in different brain areas demonstrated in the current study, it was ascertained to trace the in situ biomarker assay in these brain regions specifically.

### 3.2. Effect of ME-49 *T. gondii* Strain Infection on TNF-*α* Serum Levels

TNF-*α* levels were measured during the twelfth week of the experimental study. After ME-49 strain infection, we observed changes in TNF-*α* serum levels observed with an ELISA spectrophotometer. These changes demonstrate that the cytokine increased significantly compared to all other healthy murine models (*p* value = 0.004; Figure 2(a)).

### 3.3. Effect of ME-49 *T. gondii* Strain Infection on NF-*κβ* and IGF-1R Biomarkers

**Figure 3:**
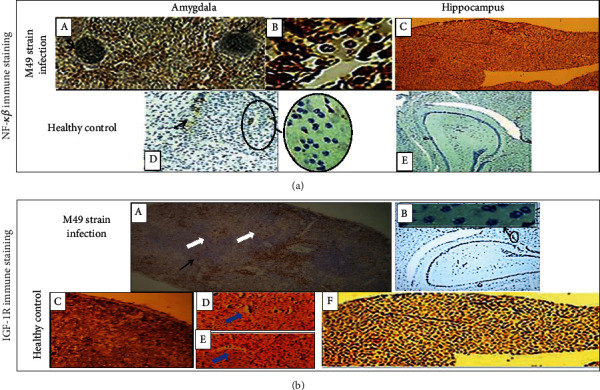
Double-labelled immunohistochemistry in the amygdala and hippocampus for the in situ biomarker assays. (a) Immune reactivity for NF-*κβ*. In the infected mice: (A) marked expression of NF-*κβ* in amygdala containing *T. gondii* cysts (black arrows) (l00x), (B) nuclear and cytoplasmic expression of NF-*κβ* in the perivascular mononuclear inflammatory infiltrates in the amygdala (400x), and (C) high expression of NF-*κβ* in the hippocampus (100x). In healthy controls: hypoexpression of NF-*κβ* in (D) the amygdala and (E) the hippocampus (40x). (b) Immune reactivity for IGF-1R. In the infected mice: (A) hypoexpression of IGF-1R around *T. gondii* cyst (black arrow) and microvasculature (white arrows) in the amygdala (40x) and (B) low evidence of IGF-1R immune staining in the hippocampus (40x). In the healthy controls: (C) high expression of IGF-1R in the amygdala (100x), (D, E) perivascular expression of IGF-1R (blue arrows) (400x), and (F) marked expression of IGF-1R in the hippocampus (100x). Spearman's rho showed that NF-*κβ* and IGF-1R area percentage and O.D. were in moderate negative correlations, -0.584 and -0.725, respectively.

Evaluating the effect of the parasitic infection on NF-*κβ* immune staining, there was marked increase while being low in disease-free murine models (Figure 3(a)). On the contrary, IGF-1R immune staining reduced sharply due to ME-49 infection and attained marked expression in healthy controls (Figure 3(b)).

**Figure 4:**
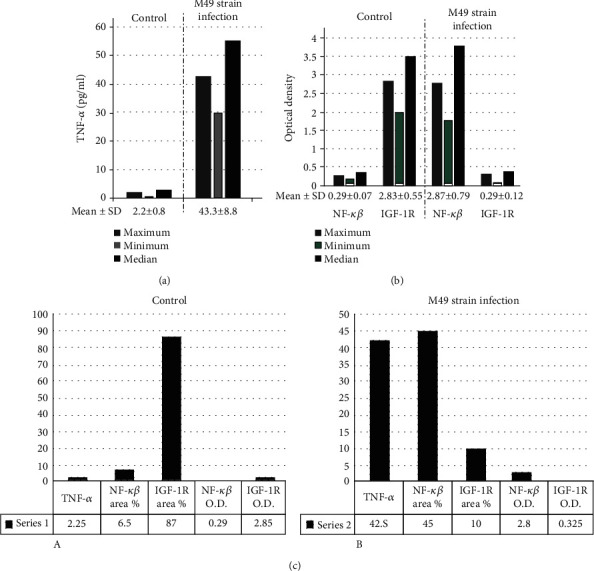
In the form of median, maximum, minimum, and median values, the bar charts in (a) and (b) show a simple descriptive statistical analysis of different biomarkers on the 12^th^ week post infection from the six chronically infected mice and healthy controls. (a) TNF-*α* (pg/ml) measured in the serum. (b) NF-*κβ* and IGF-1R intracerebral densitometry. (c) Using the mean values, the collaborative data of the biomarkers in the serum and the cerebral tissue microenvironment were shown involving TNF-*α* in pg/ml and NF-*κβ* and IGF-1R, area %, and O.D.

Statistical analysis in Figure 4(b), NF-*κβ* recorded significant higher median, maximum, and minimum values than IGF-1R in ME-49-infected mice with *p* value ≤ 0.05. On the other hand, in healthy controls, IGF-1R exhibited consistently elevated statistical values demonstrating its momentous role in the functionality of the healthy brain, whereas NF-*κβ* was hardly expressed, *p* value ≤ 0.05.

### 3.4. Correlation of TNF-*α* Serum Levels with Intracerebral NF-*κβ* Abundance at the Expense of IGF-1R

Using the Mann–Whitney *U* test and Spearman's *ρ* correlation, there were observable changes in the statistical values for the TNF-*α* cytokine in the serum samples related to the number and intensity of cells stained for NF-*κβ* (*r* value = 0.943, *p* value = 0.005). Besides, there was a strong negative correlation between the TNF-*α* and IGF-1R area percentages (*r* value = -0.841, *p* value = 0.036) and a moderate negative correlation between NF-*κβ* and IGF-1R O.D. and area percentages, *r* value = -0.7 and -0.6, respectively, and *p* value less than 0.05.

## 4. Discussion

In the present murine model, the host failed to prevent the proliferation of the tachyzoites in the acute stage, and the parasite passed to chronicity to form cysts in a process which seemed to be fully dependent on the immune system of the host [80]. Besides, *T. gondii* appeared to cross the blood-brain barrier [81, 82], to colonize in the cerebral tissues around the microvasculature. Factors beyond the selective pattern of the parasite cerebral dissemination are still vague as it spreads predominantly in the amygdala and the hippocampus [83, 84]. Mononuclear cells were abundant around *T. gondii* cysts that were most probably CD8+ T cells [80, 85] that attempt to control the *T. gondii* number of cysts [85] and act through their perforin-mediated cytotoxic activity [86–88].

Interestingly, our results showed significant elevation in the TNF-*α* serum levels which is one of the CD8+ T cell cytokines [89]. In regard to prior studies, the endogenously secreted TNF-*α* appears to be a crucial mediator of immune resistance against *T. gondii* by limiting the proliferative pattern of the early trophozoite stage [25, 89–91]. In prior studies, anti-TNF antibodies were speculated to exhibit an upsurge in tachyzoite numbers and a high mortality rate [92] due to their opportunistic nature [93]. Therefore, there should be caution against acute toxoplasmosis before anti-TNF therapy in autoimmune diseases [92–94]. Pleasantly, Leng et al. [95] reported the interference of *T. gondii* with TNF transcription at the gene's promoter site as an immune evasion process to affect the production of the proinflammatory cytokines.

TNF was accused of changing the elasticity and the strength of the neuronal synapses during various pathological conditions besides its close relation to neuronal damage [96]. Additionally, TNF-*α* together with other cytokines (e.g., IL-6) associate aging and dementia [97]. However, other authors speculated TNF-*α* to be neuroprotective in patients with dementia [98, 99].

In the infected models, NF-*κβ* showed nuclear hyperexpression in the T cells that recruited perivascular especially in the amygdala; which was speculated to be crucial in T cell activation and recruitment [28–30].

In the absence of inflammation (as in the healthy control), NF-*κβ* binds to its inhibitor protein I*κβ*, to be conserved in an inactive low expressive form [100].

Should NF-*κβ* upsurge, this would add to the implication of the parasite in the possible immunopathogenesis of dementia [101]. This is due to the fact that NF-*κβ* activation is involved in the specific inflammation of the hypothalamus seen during aging and thereby involved in the pathogenesis of dementia [102]. NF-*κβ* genuinely shares in the immune damage of the white matter; and thus, the cognitive function would be impaired [103]. In 2018, phytochemicals with an inhibitory effect on NF-*κβ* had been supposed to be a possible therapy for Alzheimer's [104].

Existence of the cyst-forming ME-49 *T. gondii* strain of the parasite was associated with IGF-1R hypoexpression in the cerebral cells especially in the amygdala. However, these low expressive patterns deprive the host cell-parasite microenvironment from (1) the modulatory and antifibrotic effects of the IGF-1 [105], (2) the protective role of IGF-1 against the inflammatory oxidative damage [106] and the hypoxic and ischemic insults [107], (3) the regeneration of the brain cells [60], (4) the normal glucose metabolism [58, 59], (5) the protection against alterations in the hormonal axis of the hypothalamus-pituitary-adrenal gland and the circulating cortisol/dehydroepiandrosterone sulfate ratios [108], and (6) the guard against atherogenicity of the blood vessels [109]. Accordingly, this might explain the observed incidence of dementia with toxoplasmosis in prior studies [6, 110].

There was a strong positive correlation between the expression of NF-*κβ* and the TNF-*α*-elevated serum level. It had been reported that TNF-*α* induces NF-*κβ* by one of two pathways, the canonical (classical) or the noncanonical. The former pathway is induced early after infection where NF-*κβ* dissociates from its inhibitory protein (I*κβ*) and translocates to the nucleus [28–30, 106]. The noncanonical NF-*κβ* pathway involves lymphopoiesis and differentiation of immune cells [106, 107] that seems to be in accordance with the recruits of T cells speculated precisely around the microvasculatures of the amygdala in the present work. Accordingly, chronic toxoplasmosis seems to be vitally regulated by the TNF-*α*/NF-*κβ* pathway.

There was a strong negative correlation between TNF-*α* serum levels and the abundance of IGF-1R in situ. Similarly, it was assumed that TNF-*α* has a regulatory effect on the expression of IGF-1 (i.e., the ligand expression) [108]. But contrary to our results, it had been deduced that in the macrophages, hyaluronic acid is capable of inducing IGF-1 expression via a TNF-dependent pathway [109].

There was a potential strong negative correlative pattern between NF-*κβ* and IGF-1R expression. Similar to our results, Mahami-Oskouei et al. [110] deduced the negative coefficient correlation of IGF-1R with the NF-*κβ*/TNF-*α* pathway in the skeletal muscles. Moreover, NF-*κβ* in ME-49 strain infection and IGF-1R in healthy controls were inspected in the perivascular spaces which question whether there is a potential cellular relationship or not.

In contrast to our results, some studies assumed that inhibition of IGF-1R attenuates expression of NF-*κβ* in the gastrointestinal tract and thus promotes healing of inflamed mucosa [111]. Interestingly, studies on the differentiation of epithelial cells estimated that the survival signal of IGF-1R/NF-*κβ* exerts a crucial nonapoptotic role by regulating caspase-3 activation [112]. Accordingly, the IGF-1R/NF-*κβ* pathway seems to be of multidisciplinary facets regarding various physiological and pathological conditions.

## 5. Conclusion

In chronic toxoplasmosis infection (ME-49 strain), (1) the cyst stage showed higher prevalence in the hippocampus and the amygdala when compared with other brain areas, (2) establishment of chronic toxoplasmosis occurs under the umbrella of TNF-*α*/NF-*κβ*, (3) TNF-*α* and NF-*κβ* are positively correlated, and (4) cerebral cells were deprived of IGF-1R expression along with this parasitic infection.

Since these findings seemed to simulate pathogenesis of dementia, we are proposing that *T. gondii* could be considered as one of its triggering factors, a matter that requires additional clinical research. Thus, we recommend further studies to identify if there are any genetic changes in the infected cerebral cells that predispose to dementia and if toxoplasmosis could be considered a risk factor in genetically susceptible patients especially during old age. Also, the available therapies are still poor in defeating chronic toxoplasmosis, another research target that may decrease the incidence of dementia. The current study introduces a pattern of biomarkers that might be experimental tools for further studies on dementia (behavior changes) using murine models infected with *T. gondii* chronic strains.

## Data Availability

Data used to support the findings of this study are available from the corresponding author upon request.
